# Reconstruction of the lncRNA-miRNA-mRNA network based on competitive endogenous RNA reveal functional lncRNAs in Cerebral Infarction

**DOI:** 10.1038/s41598-019-48435-3

**Published:** 2019-08-21

**Authors:** Jun-Bo Zou, Hong-Bo Chai, Xiao-Fei Zhang, Dong-Yan Guo, Jia Tai, Yu Wang, Yu-Lin Liang, Fang Wang, Jiang-Xue Cheng, Jing Wang, Ya-Jun Shi

**Affiliations:** 1 Shaanxi Province Key Laboratory of New Drugs and Chinese Medicine Foundation Research,Pharmacy College, Shaanxi University of Chinese Medicine, Xianyang, 712046 China; 2 The first affiliated Hospital of Hunan University of Medicine, Huaihua, 410007 China; 3 Key laboratory of Modern Prepararation of Traditional Chinese Medicine, Ministry of Education, Jiangxi University of Traditional Chinese Medicine, Nanchang, 330000 China

**Keywords:** Predictive markers, Biochemical reaction networks

## Abstract

Functioning as miRNA sponges, long non-coding RNA (lncRNA) exert its pharmacological action via regulating expression of protein-coding genes. However, the lncRNA-mediated ceRNA in cerebral Infarction (CI) remains unclear. In this study, the expression recordsets of mRNA, lncRNA and miRNA of CI samples were obtained from the NCBI GEO datasets separately. The differentially expressed lncRNAs (DELs), miRNAs (DEMis) and mRNAs (DEMs) were identified by limma package in R platform. A total of 267 DELs, 26 DEMis, and 760 DEMs were identified as differentially expressed profiles, with which we constructed the ceRNA network composed of DELs-DEMis-DEMs. Further, clusterProfiler package in R platform is employed for performing Gene Ontology (GO) and KEGG pathway analysis. An aberrant ceRNA network was constructed according to node degrees in CI, including 28 DELs, 19 DEMs and 12 DEMis, from which we extracted the core network, in which 9 nodes were recognized as kernel genes including Tspan3, Eif4a2, rno-miR-208a-3p, rno-miR-194-5p, Pdpn, H3f3b, Stat3, Cd63 and Sdc4. Finally, with the DELs-DEMis-DEMs ceRNA network provided above, we can improve our understanding of the pathogenesis of CI mediated by lncRNA.

## Introduction

Stroke is a very high risk factor for death and/or disability in the world,80% of which would be blamed for cerebral ischemic due to thromboembolic occlusion in the cerebral artery^1,2^. Half of the Cerebral Infarction(CI) affected individuals would suffer death or disability^3,4^. What’s more, the indirect cell death signals transmitted to heart would increase the risk of cardiovascular diseases roughly by three times^5^. Necropsy analyses of patients died of stroke indicate a high prevalence of coronary atherosclerosis and myocardial infarction^6,7^. However, it is still vague what the behind molecular progressions of CI are. It’s a milestone for understanding the molecular nature of CI to develop effective therapeutics.

More and more attention has been put into the regulatory network composed of long non-coding RNAs (lncRNAs), microRNAs (miRNAs) and messenger RNAs (mRNAs) to clarify the mechanisms underlying in CI. Several clinical and/or experimental studies have reported that some lncRNAs such as linc-DHFRL1-4, SNHG15, linc-FAM98A-3, SNHG12 and GAS5 take important part in the pathological development of CI^8–10^.

miRNA is a non-coding RNA composed of 22 nucleotides inhibiting the expression of a target gene by competitive binding to the response elements of microRNA. The regulating network between miRNAs and their target genes affected a variety of biological processes. miR-143-3p, miR-125a-5p and miR-125b-5p were evaluated as diagnostic biomarkers for acute CI of which the potential clinical use were also comprehensively investigated by Mahir Karakas *et al*.^11^ in 2017. Li *et al*.^12^ compiled miRNAs with the functions of regulating stroke and pre-disease mechanisms whose potential therapeutic value were further highlighted in clinical settings.

The development of CI has been proven to be involved in the competing endogenous RNA (ceRNA) hypothesis which was proposed by Salmena and colleagues in 2011. For example, Yan *et al*.^13^ uncovered the MEG3/miR-21/PDCD4 ceRNA strategy as a novel therapeutic intervention in regulating the molecular mechanisms of cerebral ischemic stroke. Chen *et al*.^14^ indicated that GAS5 acted as a ceRNA for miR-137/Notch1 signaling pathway to promote the progression of ischemic stroke form which an extensive understanding and novel therapeutic options for CI are provided.

In this paper, we retrieved RNA expression data from NCBI GEO datasets and analyzed the expression profiles between rats with middle cerebral artery occlusion (MCAO) and Sham operation. Following, we compared differentially expressed lncRNAs, miRNAs and mRNAs between the two groups. Finally, 12 miRNAs, 19 mRNAs and 28 lncRNAs were filtered out to build the lncRNA-miRNA-mRNA ceRNA network, from which we constructed a sub-network composed of 9 hub nodes including Tspan3, Eif4a2, rno-miR-208a-3p, rno-miR-194-5p, Pdpn, H3f3b, Stat3, Cd63 and Sdc4.

## Materials and Methods

### Collection of raw data

The expression recordsets of Rat mRNAs were downloaded from NCBI GEO (GSE97537) of platform GPL1355 containing 7 Sprague-Dawley rats with MCAO and 5 with Sham operation. Rat miRNAs expression data were downloaded from NCBI GEO (GSE97532) of platform GPL21572 of which containing 3 MCAO operated rats and 3 Sham operated rats. Rat lncRNAs microarray data containing 5 MCAO operated rats and 5 Sham operated rats were collected from NCBI GEO (GSE78200) of platform GPL18694. The approval from the Ethics Committee is exempt for the data deriving from the GEO database.

### Screening strategy for differentially expressed lncRNAs, miRNAs and mRNAs

The differentially expressed lncRNAs (DELs), miRNAs (DEMis) and mRNAs (DEMs) between the Sham operated and MCAO groups were determined by the two-class differential examination. The t-test was applied to filter the differentially expressed genes. The DELs, DEMis and DEMs were selected according to the *P*-values < 0.05 and fold change (log FC) > Mean (log FC) + 2*SD (log FC). In order to visualize the DELs, DEMis and DEMs, heat maps and volcano maps were generated by employing the ggplot2^15^ and pheatmap^16^ packages in the R platform.

### Prediction of target lncRNAs and mRNAs of DEMis

Firstly, the UCSC Genome Browser(http://genome.ucsc.edu/) which is proud to visualize interactions between regions of the genome were employed to annotate the lncRNAs^17^. The interaction between lncRNAs and miRNAs were predicted by LncBase Predicted v.2 of DIANA Tools^18^ and then validated by the RNAhybrid program^19^. The predicted lncRNAs of lncRNA-miRNA pairs was further filtered by matching the DELs selected before, then we can get the information of DELs-DEMis pairs.

Next, The targeted mRNA of DEMis were retrieved from MiRBase^20^, MirTarBase^21^ and Targetscan^22^. All these three miRNA references databases were highly reliable. The predicted mRNAs of mRNA-miRNA pairs was further filtered by matching the DEMs selected before, then we got the information of DEMis-DEMs.

Finally, The pairs of DELs-DEMis and DEMis-DEMs were certified.

### The construction of DELs-DEMis-DEMs network

The DELs-DEMis-DEMs network was reconstructed by aggregating all co-expression competing triplets identified above, and was visualized using Cytoscape software at the same time. All node degrees of the DELs-DEMis-DEMs network were calculated simultaneously.

### Functional enrichment analysis

Gene Ontology (GO) Biological Processes term and Kyoto Encyclopedia of Genes and Genomes (KEGG) pathway were analyzed using clusterProfiler package^23^ in R platform to make a better understanding of the behind biological mechanisms of DEMs in the DELs-DEMis-DEMs network. Then the topGO package^24^ of R platform was employed to reconstruct the GO interaction network.

### Data acquisition

The datasets analyzed in this study are available in the GEO datasets, https://www.ncbi.nlm.nih.gov/gds.

## Results

### Screening results of DELs in CI

**Figure 1:**
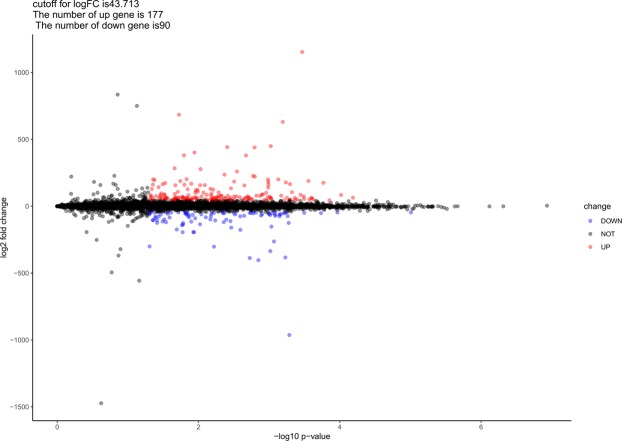
Volcano map of DELs. Up-regulated genes are represented by red spots while down-regulated genes by blue spots.

**Figure 2:**
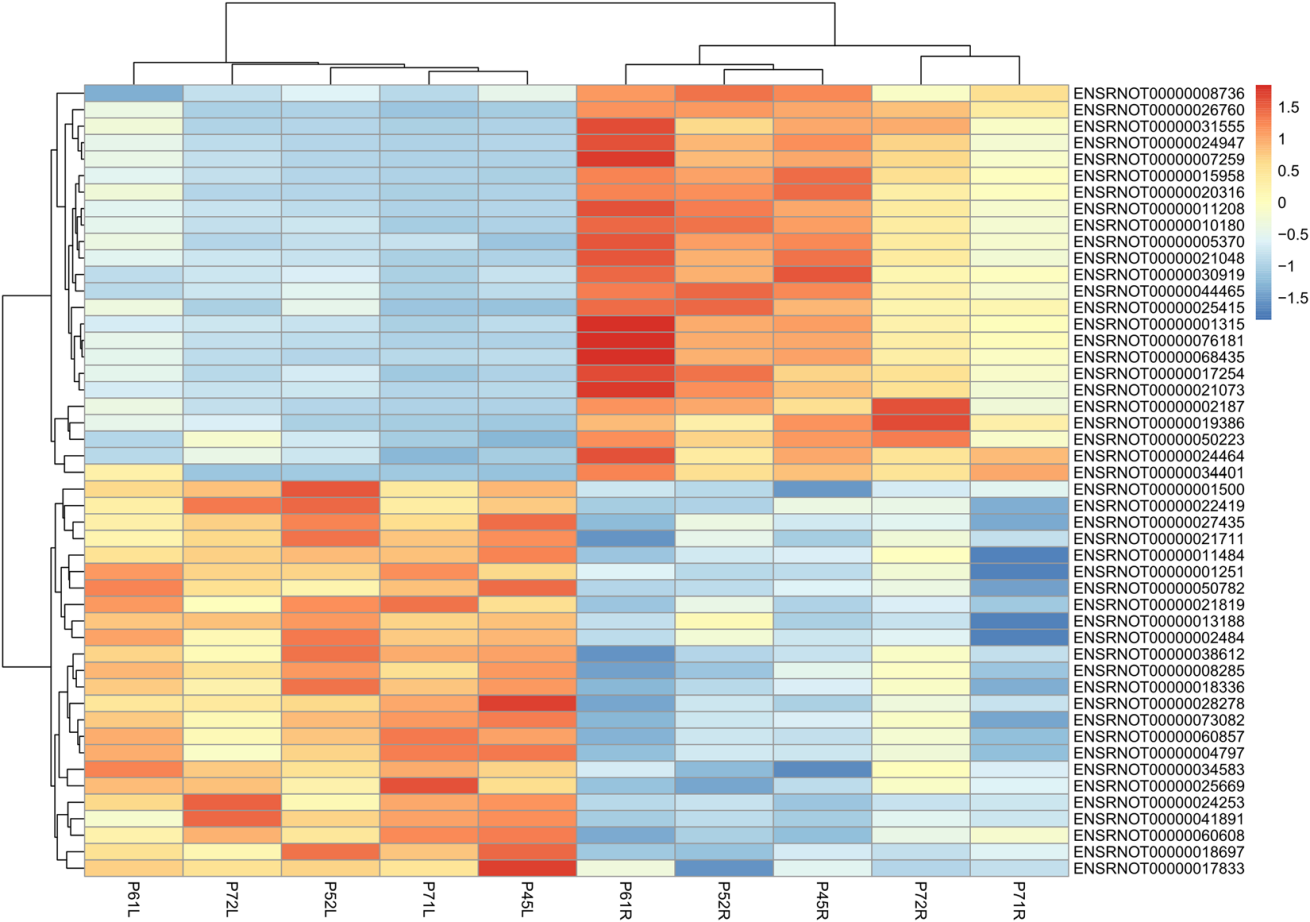
Heat map of DELs. The left 5 samples were from Sham group, the right 5 samples were from MCAO group. The color from blue to red shows the progression from low expression to high expression.

The expression levels of lncRNAs in 5 MCAO operated rats and 5 Sham operated rats were investigated in this study. According to the screening criterion described above, the cutoff for log FC of lncRNAs was 43.713, 177 (66.29%) up-regulated lncRNAs and 90 (33.71%) down-regulated lncRNAs were identified by using the limma package^25^ of R platform. In Table 1, we provided the top 25 up-regulated and 25 down-regulated ones including their symbol, logFC value, *P*-value together with FDR values. We also provided a complete file of DELs in appendix 1. In Fig. 1, a volcano map illustrating the distribution of all the DELs on the correlation of –log_10_ (p-value) and log_2_FC was exhibited. All the expression levels of lncRNAs were normalized to the sample mean. The heat map of DELs was also generated by pheatmap package in R platform as shown in Fig. 2 from which the difference between MCAO and Sham groups was visually displayed. As we can see in Table 1 and Fig. 1, the up-regulated lncRNAs are more significant than the down-regulated ones while the logFC value is relatively close.

**Table 1:** Top 50 differentially expressed lncRNAs in CI samples, half up-regulated, half down-regulated.

Name	LogFC	*P* Value	FDR	Name	LogFC	*P* Value	FDR
**Top 25 up-regulated lncRNAs**				**Top 25 down-regulated lncRNAs**			
Mfge8	64.1694	6.52E-05	1.68E-02	Stat3	−46.2758	9.83E-06	1.27E-02
Pebp1	84.4196	9.59E-05	1.91E-02	Clic4	−46.6643	1.08E-04	1.95E-02
AC136867.1	43.9340	1.40E-04	2.17E-02	S100a4	−51.4738	1.85E-04	2.38E-02
Stmn3	175.1394	1.71E-04	2.30E-02	Sdc4	−48.7466	3.19E-04	2.80E-02
Fez1	51.8946	2.38E-04	2.58E-02	Gfap	−962.8250	5.18E-04	3.16E-02
RGD1311899	63.3432	2.51E-04	2.60E-02	Atp6v0c	−124.9780	5.29E-04	3.18E-02
Scn2b	58.0023	2.53E-04	2.60E-02	Tubb6	−73.7250	5.46E-04	3.22E-02
Rabac1	49.2686	2.71E-04	2.68E-02	Hspb1	−383.3040	5.89E-04	3.30E-02
Tspan7	188.7832	2.77E-04	2.70E-02	Odc1	−49.6985	5.92E-04	3.30E-02
Sdha	54.6024	3.20E-04	2.80E-02	Pdpn	−51.9678	6.26E-04	3.38E-02
Cspg5	74.0030	3.25E-04	2.80E-02	Msn	−58.7196	6.85E-04	3.48E-02
Apoe	1152.8000	3.41E-04	2.82E-02	H3f3b	−74.9968	7.11E-04	3.56E-02
Timm8b	85.2452	3.60E-04	2.86E-02	Picalm	−61.9006	7.16E-04	3.56E-02
Tspan3	50.5604	4.00E-04	2.95E-02	Rhoc	−71.3395	8.18E-04	3.74E-02
Itfg1	47.6756	4.30E-04	3.06E-02	Tagln2	−62.7647	8.26E-04	3.76E-02
Ndufa6	63.3638	4.33E-04	3.06E-02	Arpc1b	−43.7926	8.31E-04	3.76E-02
Rnf187	104.5412	4.74E-04	3.16E-02	Fth1	−263.2750	8.53E-04	3.83E-02
Prdx6	50.0845	5.00E-04	3.16E-02	Clic1	−49.5968	9.12E-04	3.95E-02
Tuba4a	165.2066	5.05E-04	3.16E-02	AABR07054189.1	−152.7430	9.29E-04	3.97E-02
Syngr3	45.05982	5.11E-04	3.16E-02	Cd63	−335.7550	9.61E-04	3.99E-02
Mmd2	62.67402	5.60E-04	3.26E-02	Adamts1	−65.7205	9.65E-04	4.00E-02
Eif4a2	177.2346	5.79E-04	3.29E-02	Tpm4	−58.1739	1.00E-03	4.10E-02
Aco2	44.8322	6.17E-04	3.37E-02	Pfn1	−54.7670	1.02E-03	4.12E-02
Mt3	630.5722	6.42E-04	3.41E-02	Myl12a	−58.9582	1.25E-03	4.51E-02
Atp5mf	78.8416	6.76E-04	3.47E-02	Cnn3	−69.9168	1.30E-03	4.58E-02

### The DEMis screening results in CI

**Figure 3:**
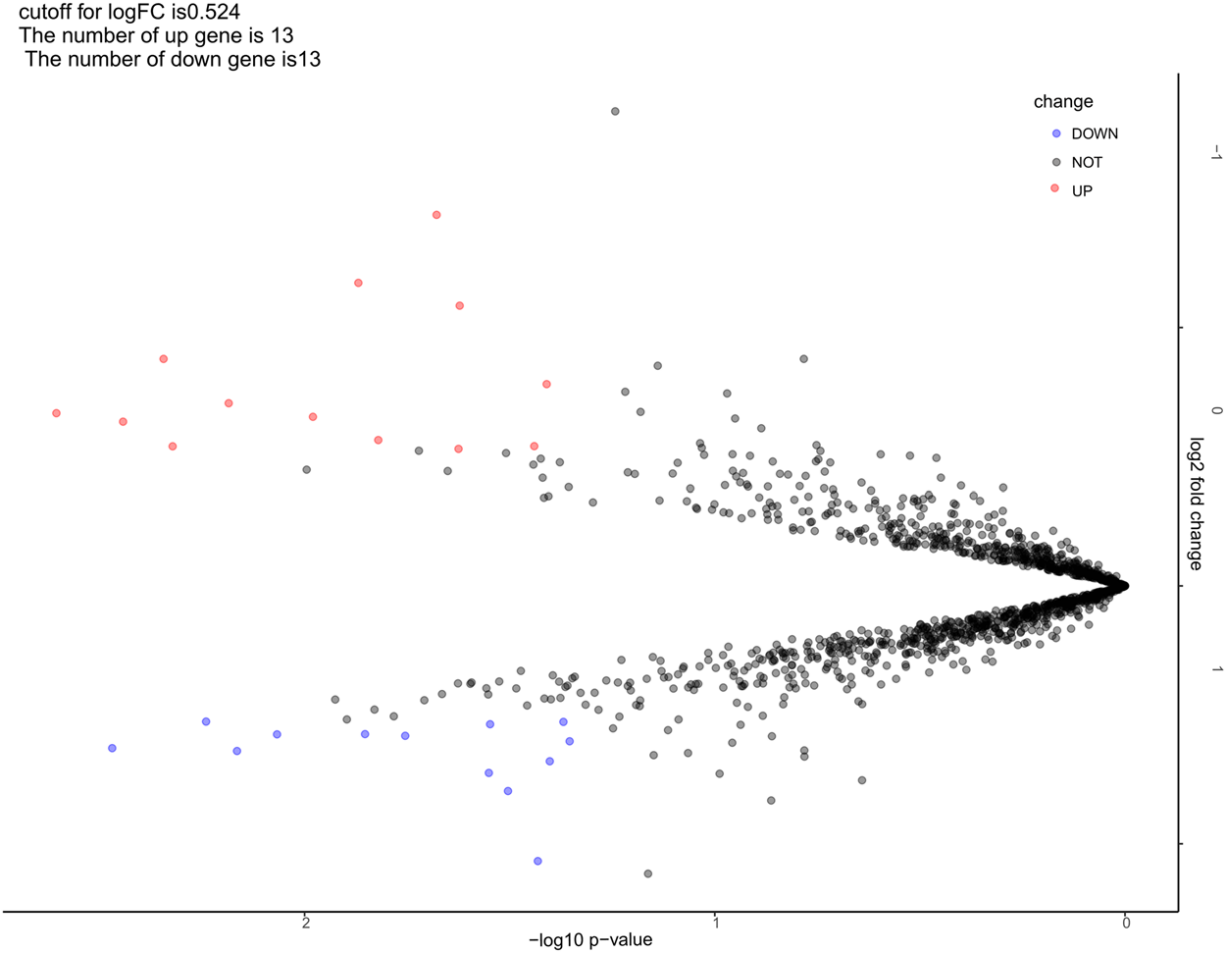
Volcano map of DEMis. Up-regulated genes are represented by red spots while down-regulated genes by blue spots.

**Figure 4:**
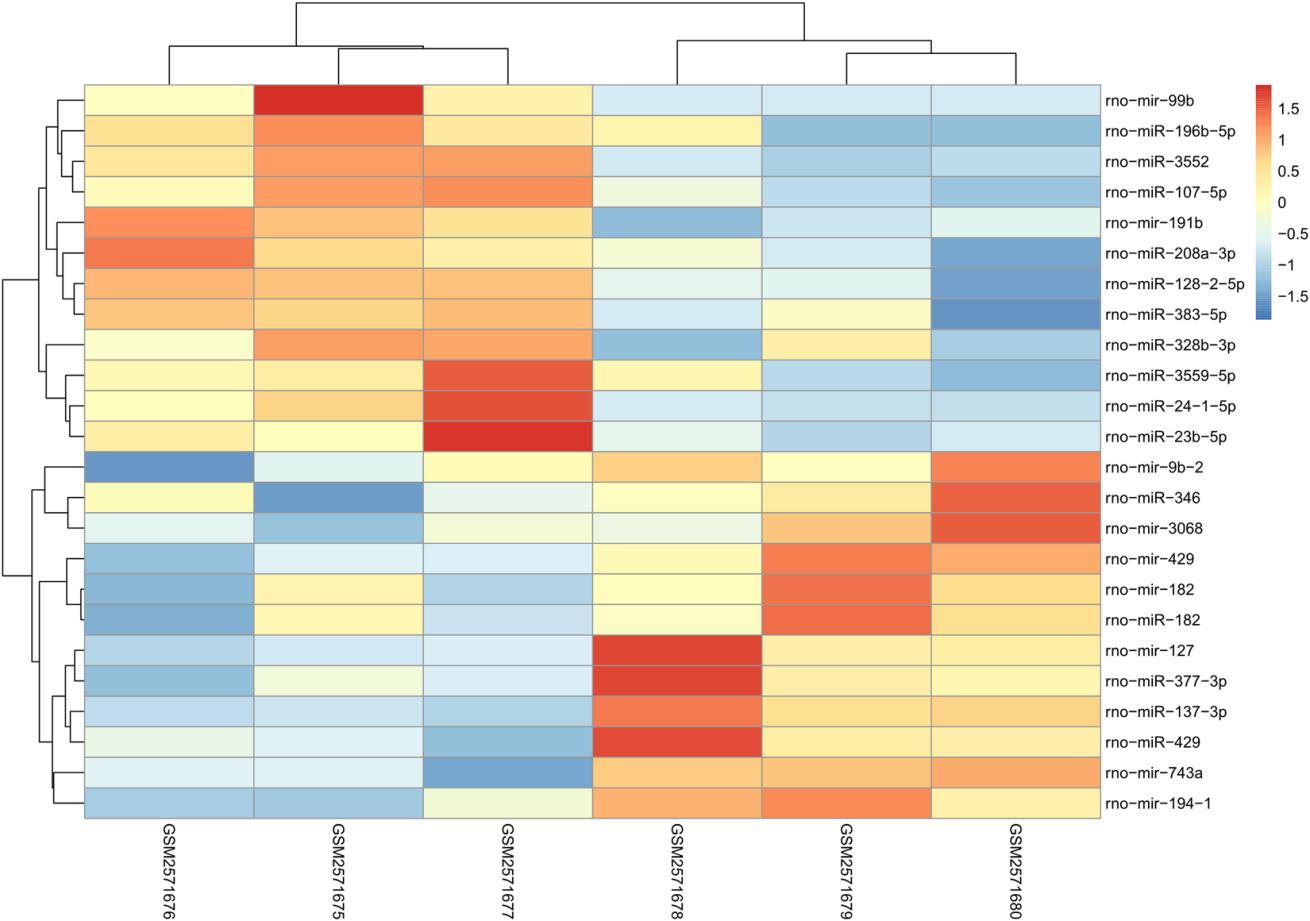
Heat map of DEMis. The left 3 samples were from Sham group, the right 3 samples were from MCAO group. The color from blue to red shows the progression from low expression to high expression.

The expression levels of miRNAs in 3 MCAO operated rats and 3 Sham operated rats were investigated. The cutoff for log FC of miRNAs was 0.524, 13 (50.00%) up-regulated miRNAs and 13 (50.00%) down-regulated ones were identified. Table 2 with their symbol, logFC, *P*-value and FDR values of all DEMis were provided. A complete file of DEMis was also settled and uploaded in appendix 2. In Fig. 3, the distribution of miRNAs on the correlation of –log_10_ (p-value) and log_2_FC was displayed by a volcano map, together with a heat map of DEMis as shown in Fig. 4 presenting the difference between MCAO and Sham groups directly. All the expression levels of miRNAs were normalized to the sample mean. There is no obvious difference between up-regulated miRNAs and down-regulated ones, as we can tell from Table 2 and Fig. 3.

**Table 2:** Top 50 differentially expressed miRNAs in CI samples, half up-regulated, half down-regulated.

Name	LogFC	*P* Value	FDR	Name	LogFC	*P* Value	FDR
**13 up-regulated miRNAs**				**13 down-regulated miRNAs**			
rno-mir-191b	0.667804	2.48E-03	9.49E-01	rno-mir-743a	−0.62925	3.40E-03	9.49E-01
rno-miR-128-2-5p	0.634881	3.61E-03	9.49E-01	rno-miR-137-3p	−0.52626	5.75E-03	9.49E-01
rno-miR-383-5p	0.878337	4.53E-03	9.49E-01	rno-mir-194-1	−0.64029	6.84E-03	9.49E-01
rno-miR-3552	0.539291	4.77E-03	9.49E-01	rno-mir-429	−0.57579	8.57E-03	9.49E-01
rno-miR-107-5p	0.706524	6.53E-03	9.49E-01	rno-mir-127	−0.5749	1.40E-02	9.49E-01
rno-miR-24-1-5p	0.65368	1.05E-02	9.49E-01	rno-miR-429	−0.58141	1.76E-02	9.49E-01
rno-miR-23b-5p	1.173112	1.35E-02	9.49E-01	rno-mir-182	−0.72532	2.81E-02	9.49E-01
rno-miR-208a-3p	0.563628	1.51E-02	9.49E-01	rno-miR-377-3p	−0.53653	2.83E-02	9.49E-01
rno-miR-3559-5p	1.436903	2.10E-02	9.49E-01	rno-miR-182	−0.79554	3.13E-02	9.49E-01
rno-miR-196b-5p	0.529474	2.37E-02	9.49E-01	rno-miR-346	−1.06718	3.70E-02	9.49E-01
rno-miR-328b-3p	1.0847	2.39E-02	9.49E-01	rno-mir-3068	−0.68017	3.96E-02	9.49E-01
rno-mir-99b	0.53982	3.63E-02	9.49E-01	rno-mir-9b-2	−0.52742	4.27E-02	9.49E-01
rno-miR-290	0.780409	3.89E-02	9.49E-01	rno-mir-139	−0.60263	4.43E-02	9.49E-01

### Results of the DEMs screening in CI

**Figure 5:**
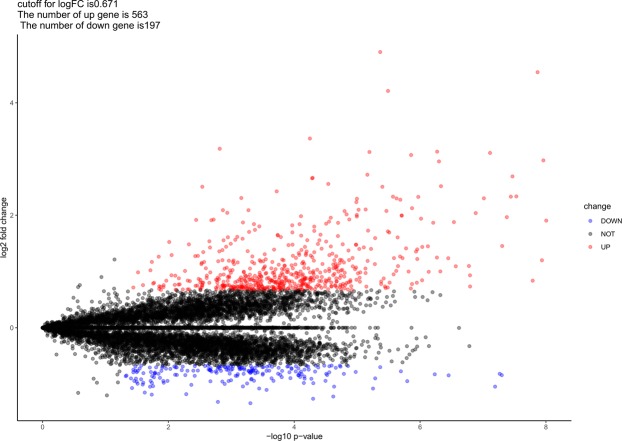
Volcano map of DEMs. Up-regulated genes are represented by red spots while down-regulated genes by blue spots.

**Figure 6:**
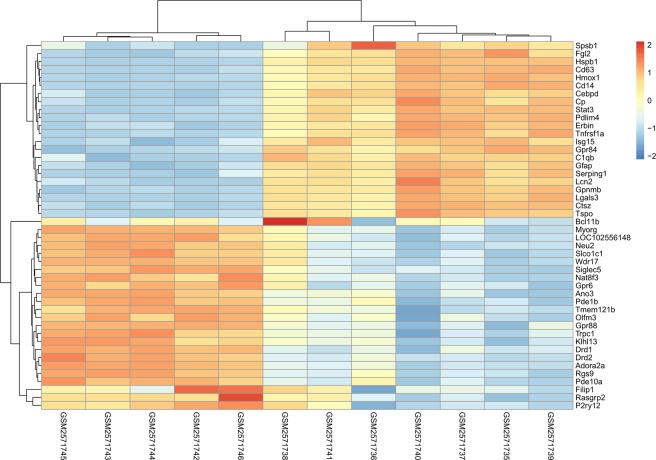
Heat map of DEMs. The left 5 samples were from Sham group, the right 7 samples were from MCAO group. The color from blue to red shows the progression from low expression to high expression.

7 MCAO operated rats and 5 Sham operated rats, whose expression levels of mRNAs were investigated in this study. The cutoff for log FC of mRNAs is 0.671, 563 (74.08%) up-regulated mRNAs and 197 (25.92%) down-regulated ones were identified. The top 25 of each are exhibited in Table 3, accompanying with their symbol, logFC, *P*-value and FDR values. We also provided a complete file of DEMs in appendix 3. Figure 5, a volcano map, illustrated the distribution of all the mRNAs on the correlation of –log_10_ (p-value) and log_2_FC vividly, based on the premise that all the expression levels of mRNAs were normalized to the sample mean. A heat map as shown in Fig. 6 was plotted to exhibit the difference between MCAO and Sham group. we can tell that the difference of up-regulated mRNAs is more significant than the down-regulated ones, reports Table 3 and Fig. 5, but the FDR value between them is fundamentally close.

**Table 3:** Top 50 differentially expressed mRNAs, half up-regulated, half down-regulated, in CI samples.

Name	LogFC	*P* Value	FDR	Name	LogFC	*P* Value	FDR
**Top 25 up-regulated mRNAs**				**Top 25 down-regulated mRNAs**			
GFAP	1.905402	9.89E-09	6.42E-05	DRD2	−0.8426	5.03E-08	8.93E-05
HSPB1	4.543782	1.36E-08	6.42E-05	ADORA2A	−0.81906	5.43E-08	8.93E-05
GPNMB	2.690464	3.41E-08	8.92E-05	DRD1	−0.84611	3.52E-07	2.82E-04
LGALS3	3.108732	7.73E-08	1.09E-04	BCL11B	−0.82749	5.85E-07	3.61E-04
CEBPD	2.301073	9.69E-08	1.28E-04	RGS9	−0.94958	1.60E-06	5.04E-04
ISG15	0.733798	1.61E-07	1.65E-04	SIGLEC5	−0.77489	2.02E-06	5.70E-04
GPR84	0.932251	1.61E-07	1.65E-04	MYORG	−0.8999	5.17E-06	8.67E-04
C1QB	1.098049	1.67E-07	1.65E-04	GPR88	−1.08078	5.60E-06	9.21E-04
CP	1.092857	2.71E-07	2.43E-04	FILIP1	−0.68048	1.40E-05	1.35E-03
STAT3	1.878466	2.90E-07	2.49E-04	PDE10A	−0.81955	1.63E-05	1.36E-03
SERPING1	1.236028	3.57E-07	2.82E-04	ANO3	−0.69081	1.84E-05	1.41E-03
LCN2	2.516615	4.66E-07	3.36E-04	TRPC1	−0.67277	1.88E-05	1.42E-03
ERBIN	1.00147	4.77E-07	3.36E-04	LOC102556148	−0.71145	2.19E-05	1.46E-03
HMOX1	2.957297	5.01E-07	3.41E-04	NAT8F3	−1.04614	2.33E-05	1.48E-03
CD14	3.131361	5.35E-07	3.44E-04	DRD1	−0.67132	2.55E-05	1.53E-03
CTSZ	1.248272	5.41E-07	3.44E-04	KLHL13	−0.68419	2.77E-05	1.57E-03
TSPO	1.867002	6.18E-07	3.69E-04	GPR6	−0.84038	3.00E-05	1.63E-03
SPSB1	0.995193	7.30E-07	3.96E-04	PDE1B	−0.81841	3.10E-05	1.65E-03
CD63	1.448319	7.52E-07	3.96E-04	RASGRP2	−0.94357	3.19E-05	1.67E-03
TNFRSF1A	1.43721	9.24E-07	4.37E-04	NEU2	−0.67389	3.33E-05	1.71E-03
ERBIN	0.844487	9.29E-07	4.37E-04	TMEM121B	−0.77907	4.67E-05	1.96E-03
FGL2	1.939632	9.65E-07	4.43E-04	SLCO1C1	−0.98522	4.98E-05	1.99E-03
CP	2.326402	1.07E-06	4.56E-04	P2RY12	−1.2622	5.02E-05	1.99E-03
Pdlim4	1.222526	1.11E-06	4.56E-04	WDR17	−0.9066	5.35E-05	2.05E-03
Fgl2	1.393555	1.11E-06	4.56E-04	OLFM3	−0.7443	5.89E-05	2.13E-03

### Functional enrichment analysis of DEMs in CI

**Figure 7:**
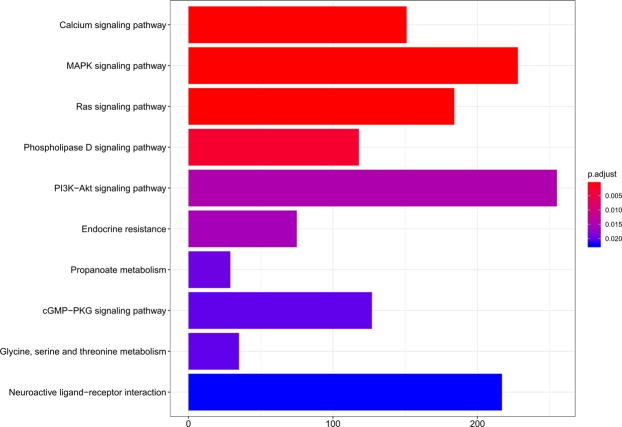
The first 10 Enriched KEGG pathways of DEMs in Cerebral Infarction. The x-axis indicates the number of DEMs participated in the pathway.

The ClusterProfiler package in the R platform was employed to execute KEGG and GO (Biological Process) analysis of DEMs in the ceRNA network, illuminating the mechanisms involved in the development of CI. 154 KEGG pathways were enriched, of which the top 20 ones were outlined in Table 4. The most important 10 pathways were shown in Fig. 7, for which bearing the most significant p-values. As we can see, Calcium signaling pathway, MAPK signaling pathway, Ras signaling pathway, Phospholipase D signaling pathway, PI3K-Akt signaling pathway, Endocrine resistance, Propanoate metabolism, cGMP-PKG signaling pathway, Glycine, serine and threonine metabolism and Neuroactive ligand-receptor interaction were involved in the pathological development of CI.

**Table 4:** Enriched KEGG pathways of MEMs in Cerebral Infarction samples.

Pathway names	*P* value	*P*.adjust	*Q* value	Count
**Top 20 enriched KEGG pathways**				
Calcium signaling pathway	6.23E-06	8.35E-04	6.74E-04	151
MAPK signaling pathway	1.42E-05	8.35E-04	6.74E-04	228
Ras signaling pathway	1.63E-05	8.35E-04	6.74E-04	184
Phospholipase D signaling pathway	8.72E-05	3.36E-03	2.71E-03	118
PI3K-Akt signaling pathway	4.90E-04	1.51E-02	1.22E-02	255
Endocrine resistance	6.27E-04	1.61E-02	1.30E-02	75
Propanoate metabolism	9.10E-04	2.00E-02	1.62E-02	29
cGMP-PKG signaling pathway	1.12E-03	2.06E-02	1.66E-02	127
Glycine, serine and threonine metabolism	1.20E-03	2.06E-02	1.66E-02	35
Neuroactive ligand-receptor interaction	1.72E-03	2.25E-02	1.82E-02	217
Cysteine and methionine metabolism	1.76E-03	2.25E-02	1.82E-02	40
Glyoxylate and dicarboxylate metabolism	1.80E-03	2.25E-02	1.82E-02	27
Rap1 signaling pathway	2.03E-03	2.25E-02	1.82E-02	158
Glycerolipid metabolism	2.05E-03	2.25E-02	1.82E-02	51
EGFR tyrosine kinase inhibitor resistance	2.30E-03	2.36E-02	1.90E-02	64
Adrenergic signaling in cardiomyocytes	3.23E-03	3.11E-02	2.51E-02	109
Alanine, aspartate and glutamate metabolism	5.47E-03	4.95E-02	3.99E-02	30
Longevity regulating pathway	6.75E-03	5.77E-02	4.65E-02	69
Glycerophospholipid metabolism	8.58E-03	6.62E-02	5.34E-02	75
Chemokine signaling pathway	8.70E-03	6.62E-02	5.34E-02	130

**Figure 8:**
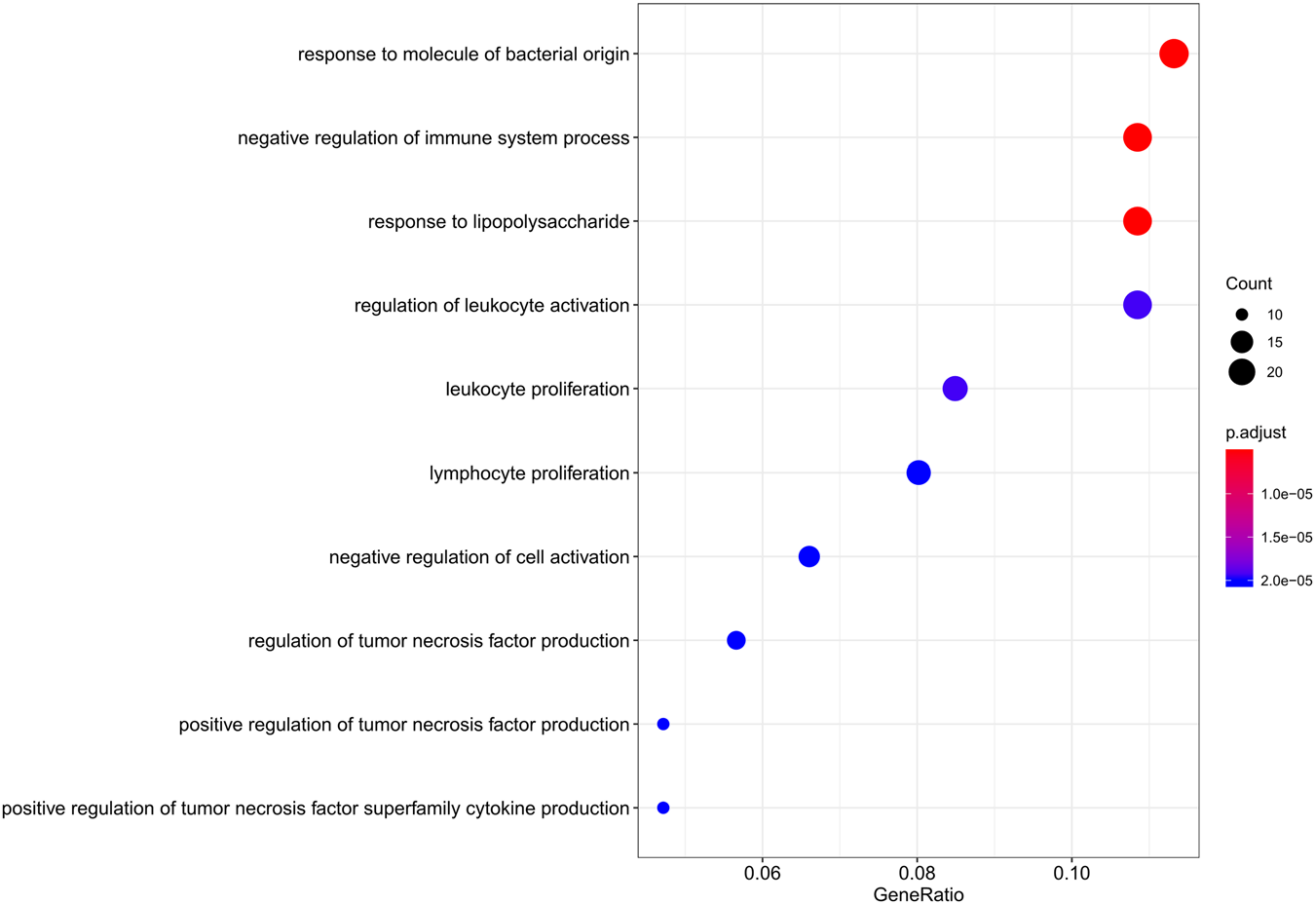
The first 10 Enriched GO BP terms of DEMs in Cerebral Infarction.

**Figure 9:**
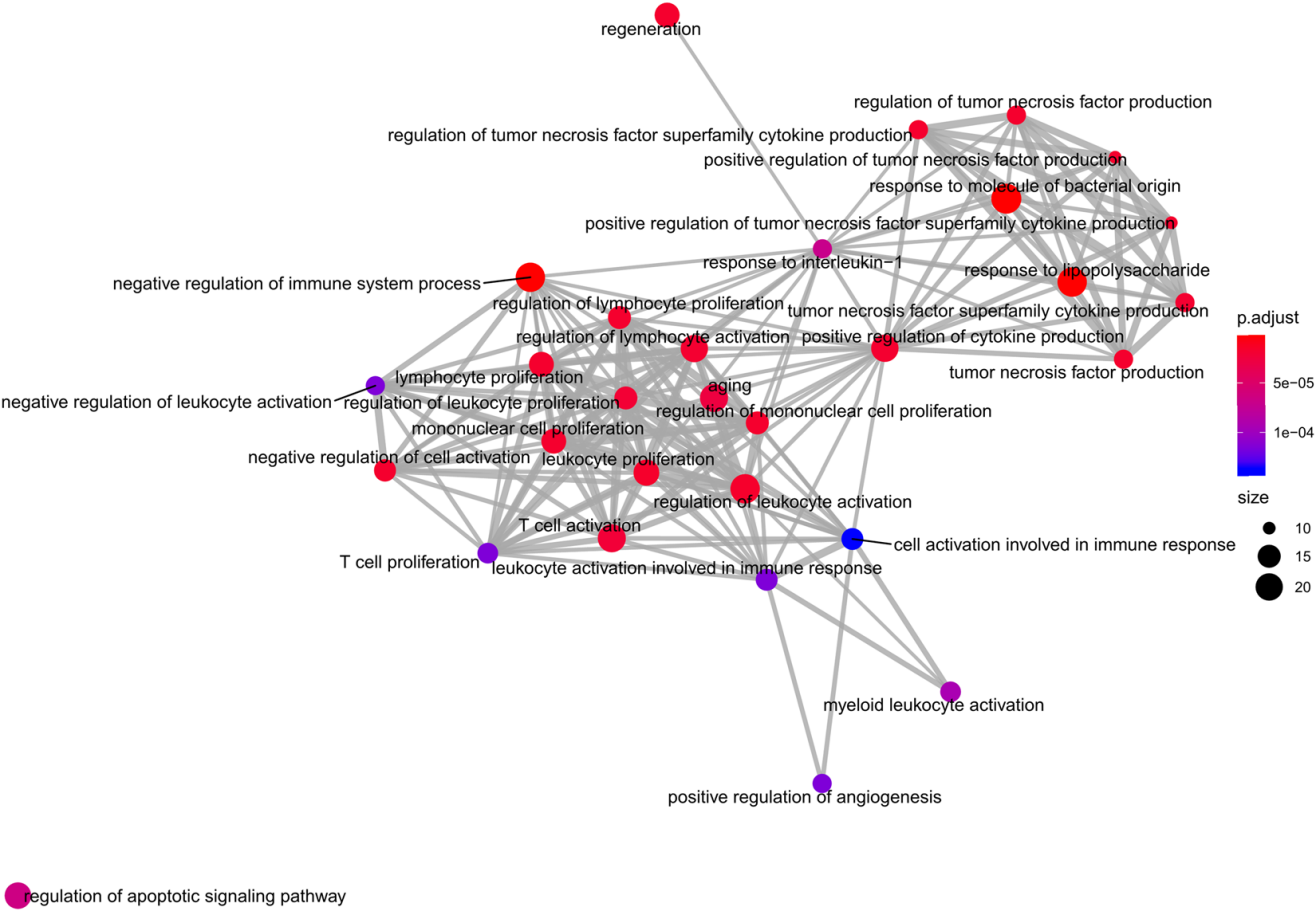
The GO interaction network of DEMs in Cerebral Infarction.

The 235 enriched GO terms in the “Biological Process (BP)” were revealed by GO analysis, including response to molecule of bacterial origin, negative regulation of immune system process, response to lipopolysaccharide, regulation of leukocyte activation, and so forth. The first 10 terms were considered as the most important ones for the most significant p-values they bearing, as shown in Fig. 8. In order to reflect the inner interactions among these GO terms, we reconstructed the GO interaction network as shown in Fig. 9.

### Reconstruction of a ceRNA network in CI

A ceRNA regulatory network of lncRNA-miRNA-mRNA was constructed to further illustrate the interaction between DELs, DEMis and DEMs which is helpful for understanding the role of lncRNAs in CI betterly.

First, LncBase Predicted v.2 of DIANA Tools was used to predict the interaction between lncRNAs and miRNAs, the interactions were further validated by the RNAhybrid program. Among all the 23 DEMis, there was 12 miRNAs interacting with 28 DELs identified by limma.

**Figure 10:**
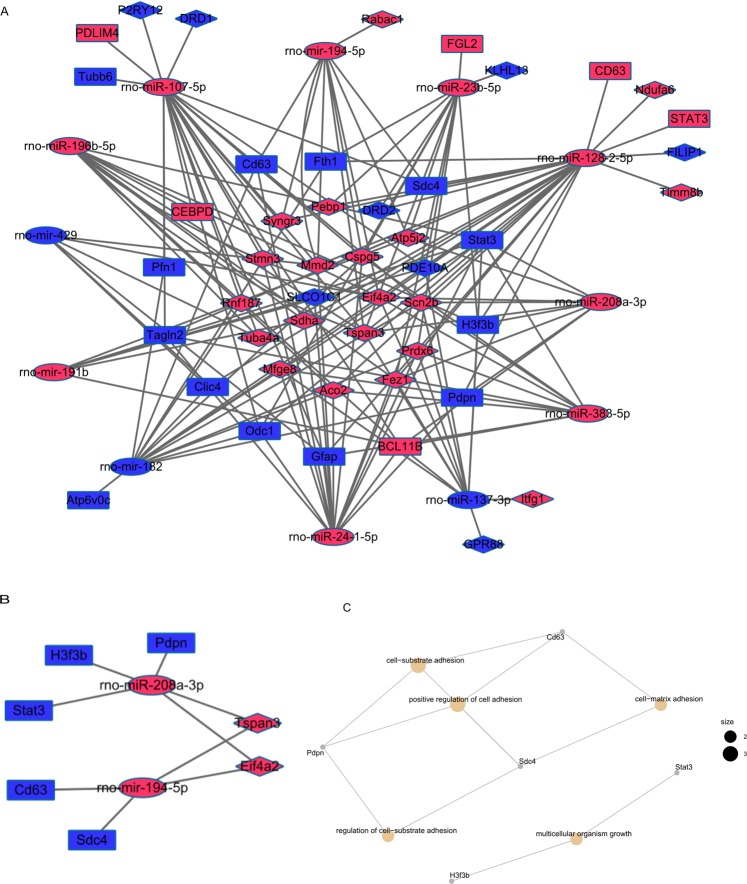
DELs mediated ceRNA regulatory network in Cerebral Infarction. (**A**) DELs mediated ceRNA network; (**B**) The sub-network; (**C**) The cnetplot of mRNAs in the ceRNA regulatory network. The red nodes indicate up-regulation expression while blue down-regulation. DELs, DEMis and DEMs are indicated by diamonds, ellipses, and rectangle, respectively.

Next, the targeted mRNA of 12 DEMis in miRNA-lncRNA pairs were retrieved from MiRBase, MirTarBase and Targetscan. We predicted that the 12 miRNAs could interact with 19 differentially expressed mRNAs identified above. Following, a ceRNA regulatory network of CI was reconstructed by incorporating 28 DELs, 19 DEMs and 12 DEMis, as shown in Fig. 10A.

The hub genes in the ceRNA network were recognized in the engaged of Cytoscape plug-in MCODE.A total of 9 nodes, including Tspan3, Eif4a2, rno-miR-208a-3p, rno-miR-194-5p, Pdpn, H3f3b, Stat3, Cd63 and Sdc4, could be selected hub nodes. The sub-network was shown in Fig. 10B. Two lncRNAs (Tspan3, Eif4a2) were found that not only had higher node degrees, but also had a higher number of lncRNA-miRNA and miRNA-mRNA pairs. This suggests that the two lncRNAs may play crucial roles in the origin and development of CI, which could be selected as the key lncRNAs. A cnetplot of hub genes indicated that the sub-network could participate in the pathological development process of CI via cell-substrate adhesion, positive regulation of cell adhesion, regulation go cell-substrate adhesion, multicellular organism growth and cell-matrix adhesion biological processes, as shown in Fig. 10C.

### Validation of key genes in the ceRNA sub-network

**Figure 11:**
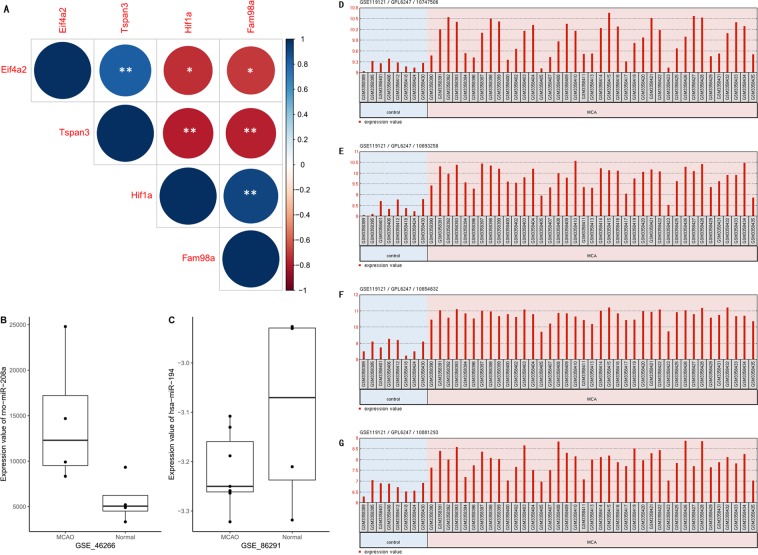
Validation of key genes of ceRNA sub-network in Cerebral Infarction. (**A**) Correlation of Tspan3, Eif4a2 with Hif1a, Fam98a; (**B**) The expression value of rno-miR-208a between MCAO and Normal group in GSE_46266; (**C**) The expression value of rno-miR-194 between MCAO and Normal group in GSE_86291; (**D**,**E**,**F**,**G**)The expression value of Stat3, Cd63, H3f3b and Pdpn between MCAO and Normal group in GSE_119121. The red color in A indicate negative correlation while blue positive correlation, size of circle indicates correlation value. *P < 0.05, **P < 0.01

Different modeling platforms were employed to verify the validation of the key genes in the sub-network of CI. lncRNA Hif1a and Fam98a were reported down-regulated significantly in rat cerebral cortex and mice brain endothelium^26,27^. Tspan3 and Eif4a2 are the two lncRNAs with up-regulated expression in the current network. A Pearson and Spearman correlation analysis on the expression among them in GSE78200 was executed to determine the validity of key lncRNAs in our finding. As shown in Fig. 11A, Tspan3 and Eif4a2 were positively correlated with each other and both negatively correlated with Hif1a and Fam98a (*P* < 0.05, *P* < 0.01). GSE46266, a GEO dataset emphasizing on the microRNAs involved in regulating embolic stroke recovery following spontaneous reperfusion in rat, and GSE86291, another GEO dataset emphasizing on microRNAs expression in Homo sapiens of hyperacute cerebral infarction were selected to determine the validity of key microRNAs. Compared to normal groups, the expression of rno-miR-208a of MCAO in GSE46266 was up regulated and hsa-miR-194 of MCAO in GSE86291 was down regulated, respectively, which is consistent with the expression patterns of the very two microRNAs in the current study, as shown in Fig. 11B,C. GSE119121 dataset was employed to verify the mRNAs, from which we can tell that Stat3, Cd63, H3f3b and Pdpn of MCAO were significantly up regulated than normal group, as shown in Fig. 11D–G, in keeping with our finding and an important backing up was provided.

## Discussion

Stroke ranks only second to heart disease for death and adult disability worldwide^28^. Ischemic stroke accounts for approximately 85% of acute cerebral vascular diseases^29^. To provide more timely reporting, only the datasets published after 2017 in GEO were included to construct the ceRNA network for illuminating the behind mechanism of CI, considering the data homogeneity requirement, GSE78200, GSE97537 and GSE97532 were filtered out to construct the ceRNA network of lncRNA-miRNA-mRNA incorporating 28 DELs, 19 DEMs and 12 DEMis. Further, a sub net-work including 9 nodes was reconstructed to propose a deeper understanding for the development of CI.

Tspan3 and Eif4a2 are the two lncRNAs in the ceRNA network. The former is a member of tetraspanin family which is widely expressed in oligodendrocytes, which forms tight junctions (TJs) of myelin sheaths in central nervous system^30^.

In this paper, we filtered miR-194 as a hub node in the sub- ceRNA regulatory of CI. Ayako Takuma *et al*.^31^ found that mir-194-1 in whole blood was down regulated significantly in their analysis of the effect of ischemic infarction. Sen Matsumoto *et al*.^32^ demonstrated that in the serum of patients after acute myocardial infarction(AMI) onset, miR-194 combined with miR-192 and miR-34a were unregulated as early as a median of 18 days. They came to the conclusion that miR-194 could serve as predictive indicators of HF. miR-208a, another microRNA in the ceRNA network and also an important member in the miR-208 family, takes important part in the development of cardiac diseases, such as myocardial infarction, hypertrophy, cardiac fibrosis and heart failure^33^. Several studies on the distribution of miRNAs in the heart, brain, kidney, lung, liver etc.^34–36^ revealed that miR-208a is exclusively expressed in heart, but here interestingly in this paper, we found the up regulation of miR-208a in blood of CI rat model which may serves as a backing for the brain-heart interaction theory.

Given the evidence of the cross regulation including hypothalamic-pituitary-adrenal axis, sympathetic and parasympathetic regulation, mircoRNAs and systemic inflammation^7^ in the brain-heart interaction after stroke, here we believe that miR-194 and miR-208a have special significance for the occurrence and development of CI which need further validation of related experiments.

Pdpn, H3f3b, Stat3, Cd63 and Sdc4 are the selected mRNAs in the ceRNA network. Kolar *et al*.^37^ suggested Pdpn as a novel cell surface marker for brain lesions with gliomas and non-neoplastic, which prevents brain injury and gliomas via normal host response. In the conclusion of Cimini *et al*.^38^, the expression of Pdpn in the infarcted myocardium were useful for identifying different cell categories, epitopes of fibrogenic and endothelial commitment. H3f3b, in charge of encoding the variant histone H3.3, is mutated in pediatric brain and bone malignancies at high frequency^39^.

Stat3, a signal transduction and transcriptive activation factor known as the signal transducers and activators of transcription family 3 protein, is, is easily activated by cerebral ischemic injury reported by several studies, implicating its vital role in the pathophysiological process of cerebral ischemia and reperfusion injury as well^40^. Endothelial Stat3 is essential for long-term recovery after stroke for its regulations on angiogenesis, axon growth and ECM-remodeling which might serve as a potential target for stroke treatment via fostering angiogenesis and neuroregeneration^41^. Phosphorylation of Stat3 at tyrosine Y705 residue is involved in microglial-mediated inflammatory processes. Pro-inflammatory cytokines after brain injury would trigger JAK kinase-induced phosphorylation of Stat3 and further regulate inflammatory process of many CNS diseases by JAK2/Stat3 pathways^29,42,43^. CRYAB/Stat3 pathway could adjust neuroinflammation, which takes important part in ischemic stroke-induced secondary cerebral injury^44^. Stat3/VEGF signaling pathway is an important pathway that affects angiogenesis and cognitive deficits in the cerebral small vessel disease^45^.

CD63 is one of platelet activation markers (CD62P, CD63, and CD40L). Tsai *et al*.^46^ demonstrated that the expression of CD63 and CD62P which were mainly enhanced in large-vessel cerebral infarction was significantly higher in acute stroke patients than in convalescent stroke and control subjects. The enhanced platelet activity would be blamed for the poor outcome and high recurrent stroke rate in large artery cerebral infarction. CD63 is also one of exosomes markers (CD63, HSP70 and TSG101) protecting remote ischemic postconditioning (RIP) on neurological damage in femoral arteries. Xiao *et al*.^47^ highlighted the importance of CD63 for CI based on their finding that CD63 was increased significantly in plasma of rat model with RIP. In the opinion of Bielecka-Dabrowa *et al*.^48^, Sdc4 serves as the only biomarkers independently distinguishing HF pts with preserved ejection fraction from reduced ejection fraction.

Based on the results of ceRNA network pharmacology analysis, we constructed a core network composed of 9 key genes including Tspan3, Eif4a2, rno-miR-208a-3p, rno-miR-194-5p, Pdpn, H3f3b, Stat3, Cd63 and Sdc4 that were thought to participate the key pathological progress of CI.

Combined with reports from existing literatures, Tspan3, a member in tetraspanin superfamily widely expressed in central nervous system, were verified to be the most important functional lncRNA regulating Tspan3/miR-194/Cd63 and Tspan3/miR-208a/Stat3 signaling pathways in CI. However, due to the lacking of direct experimental validation, the hypothesis generated above should be handled cautiously.

## Conclusion

Taken together, all the nodes in the sub-ceRNA network affect the pathological process of CI directly or indirectly. Tspan3 is the key functional lncRNA in CI regulating Tspan3/miR-194/Cd63 and Tspan3/miR-208a/Stat3 signaling pathways. However, systematic and rigorous experiments are needed to verify our findings.

## Supplementary information

Appendix 1

Appendix 2

Appendix 3
